# Neural Plasticity in Tinnitus Mechanisms

**DOI:** 10.3390/brainsci13121615

**Published:** 2023-11-22

**Authors:** Mark N. Wallace, Alan R. Palmer

**Affiliations:** Hearing Sciences, Mental Health and Clinical Neurosciences, School of Medicine, University of Nottingham, Nottingham NG7 2RD, UK; alan.palmer@nottingham.ac.uk

Basic work into neuroplasticity mechanisms in both invertebrate and vertebrate brains, followed by the development of the first animal model of tinnitus, and coupled with clinical studies of tinnitus, meant that, by 1990, Jastreboff [1] was able to propose the first comprehensive, neural model of tinnitus. This emphasized the importance of neuroplasticity and started the search for mechanisms, at the level of membrane channels, to explain neural changes and identify targets for pharmaceutical or other methods of manipulation that might be able to reverse pathological changes. The work in this field progressed in a rather piecemeal fashion in individual laboratories and medical centers until 2006, when the Tinnitus Research Initiative (https://www.tinnitusresearch.net/index.php accessed on 29 September 2023) was established in order to foster international and interdisciplinary cooperation. This led to exciting advances in the understanding of tinnitus, which were facilitated by various journals such as *Hearing Research*, *Frontiers in Neuroscience*, and *Brain Sciences*, organizing Special Issues to actively bring together the work of researchers from different backgrounds to illuminate a particular aspect of tinnitus. By September 2023, *Brain Sciences* alone had five different Special Issues that were open for submissions, looking at a variety of topics based on tinnitus (https://www.mdpi.com/journal/brainsci/special_issues accessed 25 September 2023). These follow on from previous Special Issues such as that of Neural Plasticity, which is our current focus (https://www.mdpi.com/journal/brainsci/special_issues/neural_tinnitus accessed 29 September 2023). One of the most striking features of tinnitus is that, although the matched sound level of the tinnitus that is experienced by people with chronic tinnitus (either continuous or intermittent tinnitus for more than 6 months) is relatively low, the level of distress that is experienced varies greatly. For most people, including children, tinnitus forms a slightly irritating background to their life, which they accept and find only mildly irritating, while for others, it becomes distinctly annoying or distressing, and in the worst cases, the effects of the tinnitus and its associated comorbidities are debilitating. By analyzing a population of patients who had sought online psychological help, Beukes et al. [2] were able to define four levels of tinnitus distress. In the worst cases, tinnitus can become associated with depression and suicidal thoughts. It is estimated that over 700,000 people commit suicide every year worldwide [3], and as about 1% of the population have the most debilitating form of tinnitus, this means that it could be a factor in thousands of deaths. This topic will be studied in more detail by MacDonald et al. [4].

Jastreboff’s [1] model still forms a basis for the more recent neurofunctional or Bayesian models of human tinnitus, such as those proposed by Ghodratitoostani et al. [5] or Sedley [6]. The recent work in this field emphasizes that subjective tinnitus has two components: there is the pure auditory symptom of hearing a constant but non-existent sound, and there is the distress caused by this, which can be greatly amplified when associated with other conditions—mainly anxiety, depression, and insomnia. All these models use simplified anatomical schemata, and all emphasize the distributed nature of the structures that are involved in the experience of tinnitus. They involve multiple structures in an ascending sequence, from the cochlea, via multiple brainstem nuclei, to the inferior colliculus, and then via the medial geniculate body to the core, belt, and parabelt regions of the auditory cortex. In addition, there are connections with the somatosensory, motor, limbic, and prefrontal systems. All of these different systems are connected via plastic synapses, where the activity is subject to modulating influences, which, collectively, keep the whole system in balance, even if one part becomes perturbed or altered by pathology. Tinnitus is a conscious experience with important emotional and cognitive aspects, which can only be explained at the level of the global brain systems that interact to produce conscious thought. The activity in different parts of the brain is in constant flux, and is modified by processes such as central arousal, sleep, motor effort, or sensory stimulation, as well as pathological processes such as neuroinflammation and degeneration. Cortical arousal and systems that are linked to emotions involve neurotransmitters such as serotonin and dopamine, which are very difficult to measure directly in a living subject. Thus, it is important for tinnitus research to be grounded in studies of the postmortem brain from people who experienced tinnitus in life. The work by Almasabi et al. [7] provided a rare example where it was possible to study tinnitus-related changes in the numbers of serotonergic and dopaminergic neurons, as well as evidence of neurodegeneration in brain structures that are associated with tinnitus. Reduced serotonin levels are particularly associated with depression, which is one of the main comorbidities linked to tinnitus distress, with the other being anxiety, which is particularly associated with structures such as the insular cortex [8]. The insula lies at the base of the lateral fissure and is covered by the parietal operculum, an area of the cortex that is involved in the bimodal integration of somatosensory and auditory signals. Many instances of tinnitus have a significant somatosensory component, and the review by Jaroszynski et al. [9] provides an interesting overview of evidence for the parietal operculum being one of the cortical areas involved in the tinnitus disorder. Their data implicate both the operculum and the cortex wedged between it and the main part of the insula, as being involved in tinnitus.

Another Special Issue that collected articles in 2022 (https://www.mdpi.com/journal/brainsci/special_issues/tES_application accessed on 29 September 2023) looked at the recent increase in interest in transcranial electrical stimulation as a method for the neurorehabilitation of many conditions, such as fibromyalgia and attention-deficit/hyperactivity disorder. There are a variety of techniques available, including direct current stimulation and electromagnetic induction. Tinnitus is a condition for which there is currently considerable interest in the topic of brain stimulation as a therapeutic intervention. The stimulation of the human auditory core and belt areas is difficult because they are mostly buried in the lateral fissure at a depth of 2–4 cm from the scalp. The situation is simpler in animal models of tinnitus, such as the guinea pig, with its relatively smooth cortex that allows for easy access to both the prefrontal and auditory areas. This allows controlled studies to systematically compare the effect of changing parameters such as the stimulation site, pulse frequency, and number of stimulation sessions on the strength of tinnitus symptoms over time, as shown by Amat et al. [10]. When the brain target is too deep for transcranial stimulation, then electrodes can be implanted for the deep brain stimulation of structures such as the medial geniculate body. Animal models such as the rat can then be used to study the effects of varying stimulation parameters, as described by Almasabi et al. [11]. Structures such as the amygdala and medial geniculate body are not uniform entities, despite the convenience of portraying them as such in models of higher brain function. Instead, each has multiple subdivisions with different cell types, output targets, and sources of input. For example, animal studies have shown that the medial (magnocellular) division of the medial geniculate body has a short latency input, directly from the dorsal cochlear nucleus, and projects widely to forebrain structures, including those of the limbic system; the ventral division has its major input from the central nucleus of the inferior colliculus and projects to the core auditory cortex; the dorsal division has a major input from the dorsal division of the inferior colliculus, and projects to the belt auditory cortex, while the suprageniculate division has a major input from the lateral tegmental system, and projects to the insula and forebrain motor structures. The rat medial geniculate body has a diameter of less than 2 mm, and the whole structure may be stimulated with one electrode by using a large tip and a reasonably high current. However, the human medial geniculate body has a diameter of about 1 cm, and one electrode may only effectively stimulate tissue within a few mm. Thus, detailed animal studies are still needed to describe the functional effects of stimulating distinct subdivisions of the medial geniculate body to help surgeons direct their electrodes to the most effective location. Clinical studies of cortical stimulation are more straightforward to interpret, as large, flat electrodes can safely stimulate a large cortical area. By using appropriate questionnaires, clinicians have begun to separately tease out the effects of stimulation on the basic tinnitus percept, as well as the associated conditions of depression and anxiety. These studies have been assessed in a systematic review by Labree et al. [12]. Brain stimulation requires specialized equipment and skilled operators, which means that it is unlikely to be made generally available. However, it is still generating interest, as simpler methods involving regular sessions of self-administered acoustic stimulation in an attempt to abolish the aberrant tinnitus-related neural activity have not been particularly successful. They may help some individuals, but when a double-blind, randomized, placebo-controlled trial was undertaken by Hall et al. on one of the commercially available systems, there were no significant improvements overall [13].

Despite the existence of many studies on brain stimulation, there has been, as yet, little evidence that it provides an effective method for producing a lasting benefit in people suffering from tinnitus [14], and more development is required. Similarly, the many attempts to find a pharmaceutical treatment for tinnitus have, so far, been unsuccessful. By contrast, the electrical stimulation of the cochlear nerve, whether with cochlear implants or via extracochlear stimulation, has been consistently shown to be beneficial in patients, by reducing the annoyance of tinnitus. Evidence for this has been very usefully summarized by Assouly et al. [15]. They point out that the optimal stimulation parameters have still not been identified, and suggest that, “a deeper understanding of the mechanisms involved in tinnitus suppression is needed.” One potential line for investigating these mechanisms is by comparing tinnitus to chronic pain, and many authors have emphasized their similarities. In an early such study, Tonndorf [16] postulated a role for the unmyelinated Type II fibers of the auditory nerve in an analogous way to the unmyelinated c fibers that are associated with nociceptive input in the somatosensory system. More recent research has confirmed some details of this hypothesis, and led to the suggestion that unmyelinated Type II fibers are involved in “auditory nociception” [17]. No one experiences sensations of pain in the cochlea, and the activation of these slowly conducting nerves may give rise to the perception of tinnitus instead of pain. Most people with tinnitus have either had acute noise exposure, progressive and age-related hearing loss, an ototoxic insult, or have had some form of cranial nerve damage. These conditions involve damage to the outer hair cells, which may lead to the release of ATP and cytokines as well as an increase in the spontaneous firing rate in the Type II afferents [18]. This is analogous to peripheral inflammation, which stimulates nociceptors in the somatosensory system to generate the pain percept. It is possible that tinnitus is often caused and maintained by pathological increases in the firing rate of Type II spiral ganglion neurons (SGNs), as well as their unmyelinated axons that form a small proportion (5%) of fibers in the auditory nerve. Each of the Type II afferents innervates up to 10 outer hair cells, and is thought to signal tissue damage in the same way as peripheral nociceptors. It was formerly believed that, once intractable neuropathic pain had developed, there was no need for any peripheral signal to sustain it. Similar ideas were proposed for the centralization of tinnitus. However, more recent research has shown that chronic pain such as phantom limb pain does still require a continuing peripheral signal for its maintenance [19], and this may also be true of tinnitus. Chronic neuropathic pain is unresponsive to conventional painkillers such as aspirin or opioids, and this also applies for tinnitus. However, there have been recent improvements in treatments for chronic neuropathic pain, utilizing antagonists for different classes of membrane channels. These include voltage-activated sodium channels, of which there are nine different isoforms (NaV1.1 to NaV1.9), as well as hyperpolarizing, cyclic nucleotide-dependent (HCN) channels, of which there are four isoforms (HCN1–4). The NaV1.7 channel is very important in chronic pain, as humans with a mutation that blocks its expression are unable to experience pain [20]. In the somatosensory system, blockers for the HCN2 channel [21] and the NaV1.7 channel [22] show promising effects in ameliorating various types of chronic pain. The distress that is associated with tinnitus is generated centrally, but as new drugs become available for treating neuropathic pain, there is hope that it may be possible to repurpose them to treat tinnitus as well. This provides the prospect of providing immediate, even if only temporary, relief. Some patients show spontaneous remission from the annoyance of tinnitus, and there is also the prospect of therapies that will provide more permanent relief in the future. By harnessing the increased understanding of brain mechanisms that has been produced by modern neuroscience and allying them with improved technology, it should be possible to refine the position and stimulation parameters of cochlear or cortical stimulation to allow for the permanent remediation of the neuroplastic changes that are thought to produce tinnitus. There is every chance that the current work will lead to effective treatments for the hugely damaging and distressing condition that is debilitating tinnitus.
